# Hysterectomy in benign conditions: a 20-year single-center retrospective on the development of surgical techniques

**DOI:** 10.1007/s00404-022-06821-9

**Published:** 2022-10-27

**Authors:** Paul Buderath, Rainer Kimmig, Lisa Dominowski, Pawel Mach

**Affiliations:** West German Cancer Center, Department for Gynecology and Obstetrics, University Hospital Essen, University of Duisburg-Essen, Hufelandstr. 55, 45147 Essen, Germany

**Keywords:** Hysterectomy, Robotic surgery, Benign conditions, Uterine fibroids, Surgical gynecology

## Abstract

**Introduction:**

Minimally invasive (MI) surgery has long been established as a standard for hysterectomy in benign conditions. Robotic surgery is generally seen as equivalent to conventional laparoscopy in terms of patient outcome. However, robotics might facilitate an MI approach even in complex patients, rendering laparotomy unnecessary for almost all patients.

**Materials and methods:**

We identified 1939 patients who underwent hysterectomy for benign conditions between 2002 and 2020 at the University Hospital of Essen. Peri- and postoperative data as well as patient characteristics were collected retrospectively.

**Results:**

Robotic surgery, implemented at our institution in 2010, was the most common approach (*n* = 771; 39.8%). 60.2% of all hysterectomies (1168/1938) were performed using MI techniques. However, there was a significant shift in the methods used for hysterectomy over time. While in 2002 51.4% of all hysterectomies were performed via an open abdominal approach, this percentage dropped to 1.4% in the year 2020. Accordingly, the use of MI approaches increased from 18.9% in 2002 to 98.6% in 2020. The introduction of robotic surgery in 2010 marked a significant shift towards more MI procedures. MI surgery resulted in shorter hospital stay and less postoperative complications compared to laparotomy.

On a special note, our cohort includes the largest uterus myomatous uterus in the scientific literature with a specimen weight of 54.8 kg.

**Conclusion:**

Our data support the hypothesis that the implementation of robotic surgery leads to an improved capability to perform MI surgery and avoid laparotomy in almost all patients. The known benefits of MI surgery could be confirmed.

## What does this study add to the clinical work

**Table:** 

In a retrospective analysis of 1938 patients who received a hysterectomy for benign conditions at our institution between 2022 and 2020 we were able to show a significant shift towards minimally-invasive (MI) procedures following the introduction of robotic surgery. Our data support the hypothesis that the implementation of robotic surgery leads to an improved capability to perform MI surgery and avoid laparotomy in almost all patients.	

## Introduction

Laparoscopic surgery has long been established as a less invasive and less harmful alternative to laparotomy in a multitude of indications [1]. In many surgical fields, including gynecology, minimally invasive (MI) procedures have long become standard. The benefits of MI abdominal surgery compared to laparotomy are well established in gynecology: shorter recovery time and less postoperative wound complications have been reported for benign as well as malignant conditions [2, 3].

Extrafascial hysterectomy for benign uterine conditions is the second most common surgical procedure in operative gynecology after cesarean section [4]. Indications involve uterine fibroids, abnormal uterine bleeding, endometriosis or pelvic organ prolapse (POP). Besides, hysterectomy is part of the oncological strategy in malignancies of the uterus and ovaries.

Surgical routes for the performance of hysterectomy include vaginal, open abdominal, classic laparoscopic, and robot-assisted procedures. The first open abdominal total hysterectomy was performed in 1843, while vaginal hysterectomy dates back to Sopranus of Ephesus in the year 120 AD, who removed an inverted uterus that had become gangrenous [5]. In more recent times, MI procedures such as classic laparoscopy and robotic surgery have been established. While the first conventional total laparoscopic hysterectomy was performed by Harry Reich in 1988 [6], the DaVinci surgical system (Intuitive Surgical Inc., Sunnyvale, California, USA) was approved by the United States Food and Drugs Administration (FDA) for gynecologic surgery in 2005. Since then, the robotic technique has gained popularity among gynecologic surgeons in developed countries, leading to a decrease in the use of conventional laparoscopy [7]. Potential benefits of the technology are enhanced, three-dimensional vision, increased flexibility and precision of the wristed surgical instruments and ergonomic benefits avoiding fatigue of the surgeon. A large multicenter analysis of robotic, vaginal, abdominal, and conventional laparoscopic hysterectomies performed by high-volume surgeons showed superior outcomes for the robotic technique in terms of intra- and postoperative complications [8]. Other analyses did not reveal any significant advantages of robotic hysterectomy.[9, 10]. As robotic procedures are generally associated with higher costs and longer operating times the benefit of the technique for the performance of standard procedures such as benign hysterectomy is often questioned [11].

As robotic and conventional laparoscopy seem to be largely equivalent in terms of patient outcome and both are far superior to and open abdominal approach, one of the potential advantages of robotic surgery is to facilitate the establishment of an MI approach for most patients, avoiding the increased morbidity associated with laparotomy.

The gynecologic department of the University Hospital of Essen is one of the leading centers for robotic gynecologic surgery in Europe. As such, we performed the first robotic hysterectomy in Germany in 2010 as well as the first gynecologic procedure with the daVinciXi system in 2014.

The aim of the current study was to evaluate the surgical approach to benign hysterectomy at our institution over a period of 19 years. Therefore, we examined the development of surgical techniques and the establishment of MI surgery preceding and following the implementation of robotic surgery in 2010 as well as perioperative morbidity and outcome variables. Our hypothesis was that the implication of a robotic approach on a high-volume, routine basis has led to a decrease of laparotomies, rendering an MI approach the new standard almost irrespective of patient complexity.

## Materials and methods

Patients eligible for analysis were identified by a systematic search for the ops-code 5-683 (hysterectomy) in the hospital’s own clinical information system. Primary diagnoses of all patients were documented and then filtered in order to analyze only patients with benign disease such as: uterine fibroids, abnormal bleeding, POP, benign tumor, endometriosis, or other (Chronic pelvic pain, endometrial hyperplasia, cervical intraepithelial neoplasia). Patient characteristics such as age at surgery, BMI and pre- and postoperative hemoglobin levels were assessed. Other parameters of interest were surgical data such as route of surgery, operation time, intraoperative complications and uterine weight as well as postoperative complications and length of stay. All data were documented in anonymized form. Statistical analyses were performed using SPSS Statistics^®^ Version 27 (IBM). The study was approved by the Ethics Committee of the University of Duisburg-Essen (Identifier: 16–6813-BO).

## Results

A total of 1939 patients were identified who received a total hysterectomy for benign conditions between 2001 and 2020. The most frequent indication for hysterectomy were uterine fibroids (*n* = 1092; 56.3%), followed by POP (*n* = 238; 12.3%) and abnormal uterine bleeding (*n* = 171; 8.8%). Other benign diagnoses included endometrial hyperplasia and cervical intraepithelial neoplasia (CIN) among others. Indications for hysterectomy are shown in Table 1.

**Table 1:** Indications for hysterectomy

Uterine fibroids	1092 (56.3)
Abnormal uterine bleeding	171 (8.8)
Pelvic organ prolapse	238 (12.3)
Benign tumor	83 (4.3)
Endometriosis	89 (4.6)
Other benign conditions	266 (13.7)

All values shown as *n* (%)

The mean age at the time of surgery was 49.6 years (14–90; 10.8). Mean BMI (*n* = 1245) was 27.4 kg/m2 (14.7–66.4; 6.9) and mean length of hospital stay 6.7 days (0–65; 4.7). We observed a mean decrease in hemoglobin levels of 0.68 g/dl and the mean uterine weight was 312.8 g. It has to be noted that this includes a giant uterus myomatous uterus with a weight of 54.8 kg which is to the best of our knowledge the largest specimen documented in the scientific literature with this histology. Patient characteristics are summarized in Table 2.

**Table 2:** Patient characteristics

	*n*	
Age at surgery [years]	1939	49.6 (14–90; 10.8)
BMI [kg/m^2^]	1245	27.4 (14.7–66.4; 6.9)
Length of stay [days]	1939	6.7 (0–65; 4.7)
Uterine weight [g]	1620	312.8 (10–54,800; 1428)
Skin-to-skin time [min]	1809	121.9 (9–783; 62.3)

All values are shown as mean (min–max; SD) *BMI* body-mass-index

Focusing on the route of hysterectomy, the robotic technique, which was implemented at our institution in 2010, was the most commonly used approach over the whole time period observed (*n* = 771; 39.8%). In total, 60.3% of all hysterectomies (1168/1938) were performed using MI (conventional laparoscopic or robot-assisted) techniques (Table 3).

**Table 3:** Routes of hysterectomy.

Abdominal HE	403 (20.8)
Vaginal HE	367 (18.9)
Conventional laparoscopic HE	398 (20.5)
Robotic HE	771 (39.8)

All values shown as *n* (%) *HE* hysterectomy

**Fig. 1:**
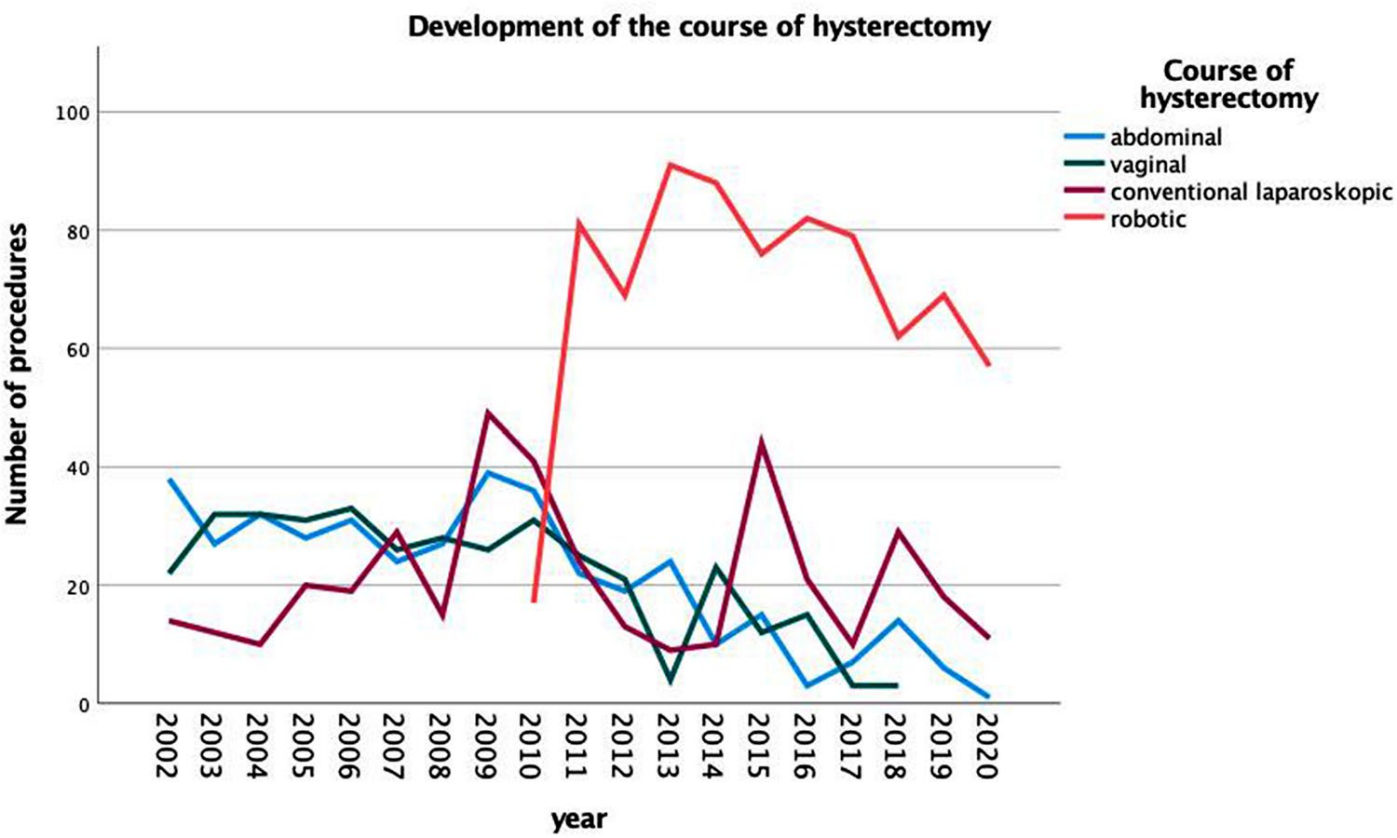
Development of the course of hysterectomy over time

However, there was a significant shift in the methods used for hysterectomy over time. While in 2002 51.4% of all hysterectomies were performed via an open abdominal approach, this percentage dropped to 1.4% in the year 2020. Accordingly, the use of MI approaches increased from 18.9% in 2002 to 98.6% in 2020. An overview of the types of procedures per year can be found in Table 4. Figure 1 offers a graphical visualization of the change in surgical approaches over the years.

**Table 4:** Routes of hysterectomy by year

	Abdominal HE	Vaginal HE	Conventional laparoscopic HE	Robotic HE	Total
2002	38 (51.4)	22 (29.7)	14 (18.9)	0	74
2003	27 (38.0)	32 (45.1)	12 (16.9)	0	71
2004	32 (43.2)	32 (43.2)	10 (13.5)	0	74
2005	28 (35.4)	31 (39.2)	20 (25.3)	0	79
2006	31 (37.3)	33 (39.8)	19 (22.9)	0	83
2007	24 (30.4)	26 (32.9)	29 (36.7)	0	79
2008	27 (38.6)	28 (40.0)	15 (21.4)	0	70
2009	39 (34.2)	26 (22.8)	49 (43.0)	0	114
2010	36 (28.8)	31 (24.8)	41 (32.8)	17 (13.6)	125
2011	22 (14.5)	25 (16.4)	24 (15.8)	81 (53.3)	152
2012	19 (15.6)	21 (17.2)	13 (10.7)	69 (56.6)	122
2013	24 (18.8)	4 (3.1)	9 (7.0)	91 (71.1)	128
2014	10 (7.6)	23 (17.6)	10 (7.6)	88 (67.2)	131
2015	15 (10.2)	12 (8.2)	44 (29.9)	76 (51.7)	147
2016	3 (2.5)	15 (12.4)	21 (17.4)	82 (67.8)	121
2017	7 (7.1)	3 (3.0)	10 (10.1)	79 (79.8)	99
2018	14 (13.0)	3 (2.8)	29 (26.9)	62 (57.4)	108
2019	6 (6.5)	0	18 (19.4)	69 (74.2)	93
2020	1 (1.4)	0	11 (15.9)	57 (82.6)	69

All values shown as *n* (%)

The introduction of robotic surgery via the daVinci^®^ system in the year 2010 offered a new surgical option for MI surgery. In the year prior to the system’s acquisition, 2009, 43.0% of all benign hysterectomies were performed using an MI approach. In 2011, the first year in which the robot was available for full 12 months, the rate was 69.1%.

The mean length of hospitalization, irrespective of surgical approach, differed significantly between the beginning of the observational period in 2002 and the last observed year, 2020 (10.7 vs. 4.5 days; *p* = 0.012). Analysis of the length of stay according to the route of surgery revealed a significant difference in favor of MI compared to non-MI methods (5.1 vs. 9.1 days; *p* < 0.001). Skin-to-skin time was shortest in vaginal hysterectomy and longest in robotic surgery. Analysis of mean BMI did not reveal any significant differences between groups. A detailed comparison of the surgical approaches is shown in Table 5.

**Table 5:** Comparison of the different surgical approaches

	Abdominal HE	Vaginal HE	Conventional laparoscopic HE	Robotic HE	*p*
Age at surgery [years]	48.6 (14–90; 11.1)	55.3 (30–90; 13.3)	47.7 (23–81; 9.1)	48.5 (18–87; 9.1)	< 0.001
BMI [kg/m^2^]	27.8 (14.7–56.6; 7.4)	27.2 (16.8–49; 5.7	26.4 (15.8–48.0; 5.4)	27.7 (16.7–66.4; 7.5)	0.093
Length of stay [days]	10.6 (3–65; 6.8)	7.6 (2–47; 4.0)	5.2 (1–16; 2.3)	5 (0–58; 3)	< 0.001
Uterine weight [g]	670.7 (18–54,800; 3173)	112 (10.4–741; 90.6)	198.5 (27–1935; 187.9)	289.1 (10–6933; 380.7)	< 0.001
Skin-to-skin time [min]	123.5 (15–447; 56.6)	96.2 (9–783; 53.8)	117.2 (41–610; 54.4)	134.2 (17–603; 67.8)	< 0.001

All values are shown as mean (min–max; SD)

Intraoperative complications occurred in 2.0% of cases (38/1938), postoperative complications were observed in 246 cases (12.7%). Most common postoperative complications were hematoma and infections (21.4% and 19.8%, respectively). 71 patients (3.7%) experienced postoperative complications of Clavien–Dindo grade 3 or higher. Two patients died in the postoperative period (0.8%). The first was a 60-year-old women with severely impaired liver function and a large serous cystadenoma of the ovary who received open abdominal hysterectomy and bilateral salpingo-oopherectomy and died following liver failure in the intensive care unit. The second was a 78-year-old, obese and multimorbid woman who was planned to receive a robotic hysterectomy for endometrial hyperplasia. Intraoperative conversion to laparotomy was necessary because the patient did not tolerate anti-Trendelenburg positioning and intraabdominal pressure caused by capnoperitoneum. The patient experienced a highly complicative postoperative period with three revisional operations due to bleeding and mesenteric ischemia. She died on the 10th postoperative day.

The rate of intraoperative complications did not differ between the routes of surgery. However, postoperative complications occurred in 24.1% following open abdominal hysterectomy, for conventional laparoscopic and robotic surgery the rates were 8.8 and 8.6%, respectively (*p* < 0.001). Rates of postoperative complications according to surgical approach are shown in Table 6.

**Table 6:** Postoperative complications according to surgical approach

	Abdominal HE	Vaginal HE	Conventional laparoscopic HE	Robotic HE	*p*
Rate of postoperative complications	24.1% (94/403)	13.1% (48/367)	8.8% (35/398)	8.6% (66/771)	< 0.001

Over the time observed, the rate of postoperative complications dropped from 21.6% in 2002 to 8.7% in 2020, mainly reflecting the shift towards MI surgery. Analyzing every surgical approach separately, no significant changes over time could be observed.

## Discussion

We present here an analysis of the perioperative outcomes in a single-institution cohort of nearly 2000 patients who underwent hysterectomy for benign conditions, covering a period of 19 years. As expected, the route of hysterectomy showed a dramatic shift towards an MI approach during the time observed. In 2020, MI hysterectomy could be performed in almost all patients with benign disease. Of note, the total number of hysterectomies showed a significant increase during the time observed, especially from 2009 onwards. This contradicts the general trend of a decrease in hysterectomies. The general development of our department in terms of case numbers and operative volume might explain a part of this effect. Moreover, as the first gynecologic robotic center in Germany, we might have profited from the relevant marketing effect of the new technology. While the use of conventional laparoscopy allowed for a MI approach for around 40% of women in 2009, the introduction of robotic surgery led to a further dramatic increase of MI procedures in the years following 2010. The rate of 98.6% MI hysterectomies in the benign cohort in 2020 shows that there are close to no limitations regarding the feasibility of laparoscopic hysterectomy using today’s technology such as robotic surgery at a trained center. During the time observed, eight different surgeons performed robotic hysterectomies pointing at the general feasibility of the technique relatively independent of individual factors. The case of the largest myomatous uterus in the scientific literature with a weight of 54.8 kg shows, that there are natural limits to every MI approach.

However, though the rate of MI procedures was almost constantly increasing since 2010, not all of these procedures were performed using the DaVinci system. In certain years, a conventional laparoscopic approach was used for up to almost 30% of patients (2015). The reasons for this lay in the limited availability of the DaVinci system due to a shared use with other disciplines such as urologic and general surgeons as well as an increase in robotic procedures in malign conditions. However, the trend towards MI surgery remained unbroken. The fact that procedures could be switched from robotic to conventional laparoscopy in times of limited robotic capacities points at the general equivalence of the surgical skills required by the different MI approaches. Surgeons who are trained to perform even complex benign hysterectomies robotically might very well be able to perform the same kind of surgeries using conventional laparoscopy, benefitting from their experience in robotics. However, robotic hysterectomy remained the method of choice for the majority patients with benign disease from 2011 onwards, pointing out the immense importance of the technology for the establishment of MI surgery as a standard of care.

The advantages of minimally invasive surgery regarding the avoidance of complication and reduction of hospital days are well documented [12]. Our data confirm these observations in a real-life setting. Length of stay was less than half as long after MI surgery compared to laparotomy. Besides the surgical approach, general developments in fast-track surgery as well as the general trend towards early postoperative discharge might have contributed to this finding.

Postoperative complications were reduced to around a third by choosing a MI approach. This is in line with the available literature [2, 3]. It has to be pointed out that all data presented here were collected retrospectively. Thus, the rate of complications reported depends on the quality of documentation as well as the observational period. Since patients after MI surgery have much shorter hospital stays, less complications could be observed. On the other hand, the implementation of digital patient charts and the increasing standards regarding documentation might contribute to more detailed assessment of the postoperative course in recent years.

In recent years, robotic surgery has gained relatively quick acceptance in the performance of gynecological operations, including simple hysterectomy [12]. A study from 2013 showed that the use of robotic hysterectomy increased by almost 1000% between 2007 and 2010, while the frequency of conventional laparoscopic hysterectomy increased at a much slower rate [13]. Especially in obese women, the benefits of robot surgery compared to conventional laparoscopy have been demonstrated [14]. In addition, in the case of large uterus size [15–17], reduced blood loss, reduced postoperative pain and shorter hospitalization with higher surgery costs were shown. However, most of these studies are not randomized and the results are heterogeneous. Despite the lack of randomized data supporting the use of the robot in hysterectomy with benign indications, many surgeons choose this method as an easy to master and quickly adaptable surgical tool [12].

So far, in most cases, three main aspects of robotic surgery were compared with other surgical routes, including surgery time, estimated blood loss and length of hospitalization after surgery. However, since cost-effectiveness is the greatest constraint on robotic surgery, future studies must include cost assessments, including calculations of hospitalization length, postoperative complications and return to daily life and convalescence. Based on current knowledge, the choice of surgical approach in case of gynecological surgery should be individualized based on the patient’s disease, the surgeon’s experience and the availability of robotic equipment. It has been shown that robotic surgery can be advantageous in comparison to open abdominal, vaginal and conventional laparoscopic approach in the hands of high-volume surgeons [8]. However, many non-medical factors, such as the economic conditions of the hospital or the willingness of surgeons to use modern technology also play a role in the choice of selection of surgical route.

In conclusion, our data document the shift from mainly open abdominal surgery to an almost exclusively MI approach at our institution since the turn of the millennium. The implementation of robotic surgery marks a significant turning point towards an almost complete avoidance of laparotomy for hysterectomy in benign conditions. The known benefits of MI surgery could be confirmed in our collective. While it does not seem to matter if MI hysterectomy is performed using conventional laparoscopy or robotic surgery, these findings suggest that MI hysterectomy should be the standard of care for benign disease.
